# Proactive community support tailored to holistic needs: A cohort study

**DOI:** 10.1002/cam4.1709

**Published:** 2018-08-13

**Authors:** Austyn Snowden, Jenny Young, Jan Savinc

**Affiliations:** ^1^ School of Health and Social Care Edinburgh Napier University Edinburgh UK

**Keywords:** cancer, caring responsibilities, follow‐up, needs assessment, unemployment

## Abstract

**Background:**

It is increasingly internationally recognized that a cancer diagnosis impacts on people practically and financially as well as physically and psychologically. It is less clear what to do about this. This study introduces an original community service designed to mitigate this wider impact. Nonclinical “link officers” use holistic needs assessment (HNA) to help newly diagnosed people identify and quantify the severity of their physical, psychological, practical, financial, and social concerns. A care plan is then agreed, usually involving community interventions from partner agencies. Following intervention, assessment is repeated. The primary aim of this study was to establish whether there was a significant difference between initial assessment and follow‐up, postintervention. Secondary aim was to identify potential predictors of increased levels of concern at baseline and follow‐up.

**Method:**

Pre‐ and postintervention observational cohort study. Paired *t* test examined the difference in mean (SD) concern severity between baseline and follow‐up. Multiple linear regression models were computed to hypothesize potential predictors of initial concern severity and severity change.

**Results:**

The service saw 2413 people 2014‐2017. Participants identified average 5.5 (4.7) concerns, financial concerns being most frequent. Mean severity at baseline was 7.12 (out of 10) (2.50), reducing to 3.83 (3.49) post‐treatment, paired *t*(4454) = 64.68, *P* < 0.0001, reduction of 3.31 (95% CI 3.21‐3.41). Factors associated with higher initial concern included unemployment and caring responsibilities. Unemployment was also associated with a smaller reduction of concern severity at follow‐up.

**Conclusion:**

Patient level of concern went from a level associated with specialist referral to a much more manageable level. This original finding is internationally significant because it extends Khera et al's (2017) “provocative idea” that all patients should be screened for financial problems to show that they can be helped with *all* their concerns. This article describes a successful, transferable model of community care.

## 1 INTRODUCTION

International policy states that people newly diagnosed with cancer should have their psychological needs screened for.^1^ However, patients are less likely to be screened for other concerns, such as practical, social, and financial concerns.^2^ One of the main reasons is that there is no consensus on how to support people with these issues.^3^ Ideally, services would work together proactively to meet individual need, be it physical, psychological, practical, or spiritual^4^, ^5^ but there is little evidence to guide commissioners of such services. Research designed to evaluate a service constructed specifically to meet these needs is therefore timely.

### 1.1 Background

Cancer care has traditionally been carried out in an outpatient setting. Patients are known to be generally satisfied with clinical aspects of their care,^6^, ^7^ but less pleased with the nonclinical elements.^8^ They report poor communication in relation to their practical and emotional concerns and have been dissatisfied with the lack of financial advice and support available.^9^, ^10^ They recognize that the clinical setting may not be the best place to receive such support, so they can be reluctant to disclose psychosocial concerns there, perceiving the staff to be too busy.^11^ Patients do not want to be seen as being demanding or “difficult”,^12^ even when in considerable need,^13^ so there is a global unmet need for information, social, emotional, and psychological support.^14^ Many people with financial problems and practical issues such as transport, parking, and getting around generally lack the support they need.^15^

The financial burden of a cancer diagnosis is becoming better understood.^2^ In UK, poorer people are at risk of losing their homes.^16^ In United States, around 9% people were uninsured in 2016; people who are less likely to seek early treatment, worsening prognosis, and increasing overall cost.^17^ Financial burden is associated with poorer quality of life,^10^ so it follows that if people at risk of struggling financially could be identified earlier, and then, they could be helped quicker, potentially avoiding or mitigating negative impact on quality of life.

**Figure 1:**
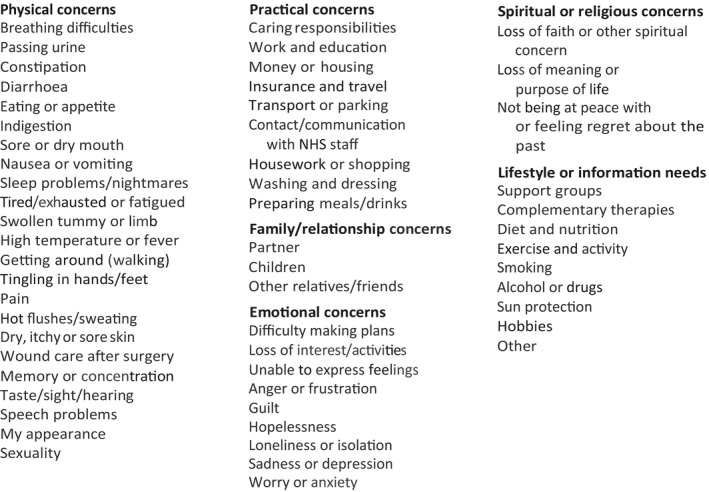
Holistic needs assessment

“Improving the Cancer Journey” (ICJ) was designed to proactively help newly diagnosed patients with all these issues. ICJ is a community‐based interdisciplinary cancer service led by the local authority (the legislative body that governs the city) and is the first of its kind in the UK. ICJ invites patients to take part in a “holistic needs assessment” (HNA), a structured assessment designed to identify a patient's individual concerns in order to help them. It consists of 57 items covering physical, psychological, social, financial, spiritual, and practical concerns (Figure 1). Once patients have identified and rated their concerns, action is then taken to mitigate them by constructing a care plan. The care plan usually takes the form of providing relevant information, signposting, or referral to various partner agencies, with a focus on enabling people to help themselves wherever possible.^18^

The HNA was derived from the distress thermometer (DT).^19^ They include the same problems and both use a 0 to 10 scale to identify severity. A major difference is that rather than generating an overall score of general distress in the case of the distress thermometer, HNA records an individual score for every concern identified. If the severity of concerns is seen to reduce between initial and follow‐up visit, then it would be reasonable to conclude that ICJ may have made a contribution toward the improvement. This study is designed to test this and identify any other factors that may explain the data. The primary aim of this study was therefore to establish whether there was a significant difference between initial assessment of concern and follow‐up scores. However, any observed change is highly likely to be multifactorial. Having comorbidities or mental health issues may make a difference for example,^20^ as could age, gender,^21^ type of cancer,^22^ and socioeconomic status as well as time^10^ and type of intervention. Secondary aim of this study was therefore to identify potential predictors of (a) increased levels of concern at initial visit and (b) change in severity scores at reassessment.

## 2 METHOD

### 2.1 Design

Pre‐ and postintervention cohort study following STROBE guidelines for reporting.^23^

### 2.2 The intervention

#### 2.2.1 Invitation

The Information Services Division (ISD) is a division within the National Health Service (NHS), providing health information, health intelligence, and statistical services. Where someone has just received a diagnosis of cancer in Glasgow City area, ISD posts them a letter of invitation on behalf of the service. The letter invites patients to contact the ICJ service and arrange an appointment with a “link officer” at a place of the patient's choosing. Around 50% people take up the offer.

#### 2.2.2 Link officers

ICJ link officers are city council employees, not health care professionals. The council currently employ six full‐time link officers. When they first join the service, link officers have a 3‐month induction period where each officer becomes familiar with their role and completes a range of training. Currently, all officers are, or are working toward, being accredited with a Level 3 Scottish Vocational Qualification (SVQ) in healthcare support to reflect their competencies in this area. Level 3 SVQ is a vocational qualification academically equivalent to graduate diploma level, or second year of baccalaureate degree.^24^

#### 2.2.3 The assessment

Over 90% first assessments happen at the patient's home, with the remainder conducted in libraries, council buildings, and hospitals. On meeting, the link officer goes through the HNA with the patient. Each of the 57 concerns are discussed and if relevant to the patient, given a score between zero to 10. Results are recorded by the link officer on electronic tablet, to be transferred later to a secure central database used to record all interactions within the council. The purpose of the HNA is to identify people's most serious concerns.

Once identified, a care plan is coconstructed between the patient and link worker, designed to mitigate the most serious concerns. Often the plan takes the form of signposting to a particular agency, or referral to relevant services. ICJ works in partnership with over 200 local agencies, and all referrals are managed by the link officers. All referrals, and whether patients subsequently attend or not, are recorded centrally along with their assessment and care plan. After the initial visit, the patient receives a letter from ICJ detailing the agreed care plan and a summary of the discussion.

The initial assessment process usually takes just over an hour, averaging 68.6 (20.5) minutes. At an agreed time, allowing for completion of the care plan, the link officer telephones the patient to review them. This call usually happens between four to five months after initial contact, although the timing depends on individual circumstances, and of course, patients can contact ICJ again if needed within this time. At this follow‐up appointment, HNA is repeated and concern scores are reassessed.

### 2.3 Participants

This study included all individuals over 18 years who agreed to take up the offer of ICJ, and who completed an initial HNA in the period between February 14, 2014, and July 21, 2017. People incapable of consent were excluded.

### 2.4 Ethics

The study was reviewed and approved by the West of Scotland Research Ethics Committee (WS/15/0166) and Edinburgh Napier University School of Health and Social Care Ethics Committee.

### 2.5 Hypotheses

The primary aim of this study was to establish whether there was a significant difference between initial assessment of concern and follow‐up. Hypothesis one was therefore:

1. There will be a significant decrease in mean concern scores between baseline and follow‐up

The secondary aims were twofold:

- to identify potential predictors of high levels of concern at initial visit,
- to identify potential predictors of change in severity scores at reassessment.

### 2.6 Analytic plan

All data were imported into R package for statistics, checked for normality and homogeneity of variance. For the main hypothesis, paired *t* test was run to ascertain the difference in HNA scores between initial visit and follow‐up, postintervention.^25^ For the secondary aims, demographic and diagnostic variables associated with initial concern levels were tested in multiple linear regression models to identify likely predictors of high level of initial concern. The same process was undertaken with variables associated with the change in severity between assessments, to identify potential predictors either facilitating or suppressing change.

## 3 RESULTS

### 3.1 Demographics

In 2017 a total of 2413 people had used the service, 1286 (53.3%) females and 1127 (46.7%) males, with 60.8% coming from areas in the most deprived quintile of the Scottish population‐adjusted multiple deprivation index.^26^ Mean age was 63.5 years (12.9), ranging between 22 and 100.

The top four cancers (lung, breast, prostate, and bowel) accounted for 58.5% of all diagnoses, with 83 other cancers accounting for the rest. At least one comorbidity was reported by 53.7% of patients; 14.6% noted the presence of mental health problems, and 8.0% reported a mental health diagnosis as a comorbidity. A total of 45.5% were partnered, and 79.1% described themselves as white Scottish. Just over half (1258, 52%) were retired, with 515 (21%) in work and 613 (25%) unemployed. For detailed descriptive data please see Data S1.

**Figure 2:**
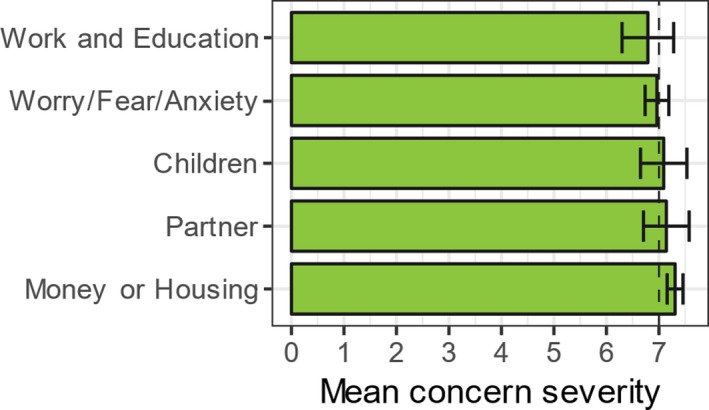
Top five concerns at initial assessment. Error bars represent 95% CIs of concern severity. Dashed line shows DT threshold value at 7

Participants on average identified 5.3 (4.9) concerns at initial appointment. The top five concerns were “money or housing,’ “partner,” “children,” “worry/fear/anxiety,” and “work and education” (Figure 2). The average time between first and follow‐up assessment was 158.77 (129.64) days.

All patients were followed up but not all were necessarily reassessed using HNA. For example, where concern levels had been low at initial assessment and remained so on telephone follow‐up, assessment was not repeated. Others remained in contact with ICJ at time of writing. Analysis of change in severity scores was therefore based on the patients who had been revisited and reassessed with HNA (N = 1142).

### 3.2 Analyses

**Figure 3:**
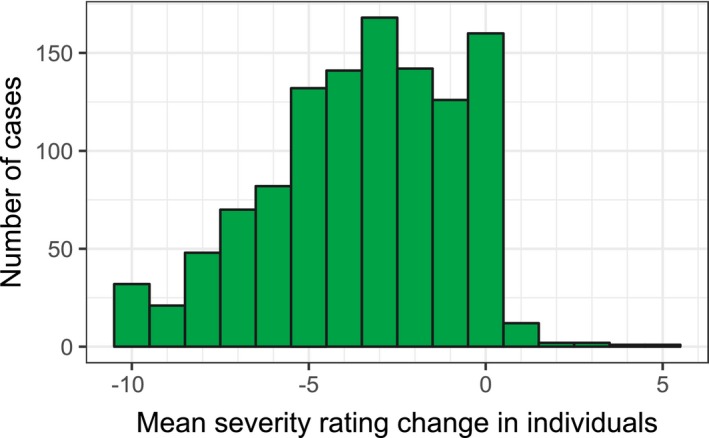
Distribution of mean severity difference scores between initial assessment and follow‐up for individuals. Negative scores correspond to a reduction of severity

The scores were not normally distributed (see Figure 3). However, the sample size was large enough to warrant using *t* test as it is robust to violations of normality as long as sample sizes are large enough.^25^ The primary aim of this study was to establish whether there was a significant difference between initial assessment of concern and follow‐up. The mean severity of all concerns at first assessment was 7.12 (2.50), reducing to 3.83 (3.49) at follow‐up, paired *t*(4454) = 64.68, *P* < 0.0001, a significant reduction of 3.31 (95% CI 3.21‐3.41).

Bivariate analyses were then used to explore the associations between demographic factors, diagnostic factors, and the outcome variables—mean initial severity of concerns and the mean change of concern severity between assessments. Correlations were computed for continuous variables (Tables 1 and 2), and ANOVA *F* tests were computed for categorical variables (Tables 3 and 4).

**Table 1:** Continuous variables’ association with mean severity at initial assessment. Age was approximately normally distributed; the number of comorbidities and deprivation vigintiles were severely left‐skewed, so Spearman's rank correlation was used to test for association with mean concern severity

Variable	N	M	SD	*r*_Pearson_/*ρ*_Spearman_	*P*
Age (22‐100)	1907	63.4	12.9	*r* = −0.073	0.0013**
Number of comorbidities at initial assessment (0‐5)	2236	0.99	1.16	*ρ* = 0.021	0.31
Deprivation vigintile (1‐20)	2229	5.23	5.06	*ρ* = −0.063	0.0029**

*indicates *p* < .05, **indicates *p* < .01, ***indicates *p* < .001

**Table 2:** Continuous variables’ association with mean severity difference. Age was approximately normally distributed; the number of comorbidities, deprivation vigintiles, and days elapsed between assessments were severely left‐skewed, so Spearman's rank correlation was used to test for association with mean severity decrease

Variable	N	M	SD	*r*_Pearson_/*ρ*_Spearman_	*P*
Age (23‐95)	993	62.0	12.3	*r* = −0.017	0.59
Number of comorbidities at initial assessment (0‐5)	1058	1.04	1.18	*ρ* = 0.030	0.33
Deprivation vigintile (1‐20)	1057	5.06	5.04	*ρ* = −0.055	0.073
Days elapsed between initial assessment and review (7‐885)	1107	146.3	122.1	*ρ* = −0.067	0.027*

*indicates *p* < .05, **indicates *p* < .01, ***indicates *p* < .001

**Table 3:** Categorical variables’ association with mean concern severity at initial assessment. Cancer diagnoses were individually compared against the grand mean across all diagnoses

Variable	N	Mean	SD	*P*
Sex				
Female	1209	6.71	2.29	
Male	1032	6.30	2.43	0.000059***
Cancer history				
No	1758	6.42	2.39	
Yes	427	6.91	2.24	0.00012***
Cancer diagnosis				0.0087**
Bowel	242	6.16	2.54	0.013*
Breast	367	6.80	2.14	0.012*
Lung	505	6.43	2.48	0.32
Prostate	208	6.34	2.44	0.27
Other	914	6.59	2.32	0.24
Unemployment				
No	1654	6.37	2.37	
Yes	587	6.96	2.30	<0.00001***
Financial problems				
No	1257	6.51	2.35	
Yes	984	6.54	2.39	0.74
Mental health problems				
No	1903	6.45	2.34	
Yes	338	36.91	2.49	0.0011**
Caring responsibilities				
No	1861	6.47	2.38	
Yes	380	6.77	2.29	0.026*
Mobility problems				
No	909	6.29	2.30	
Yes	1331	6.68	2.40	0.040*

*indicates *p* < .05, **indicates *p* < .01, ***indicates *p* < .001

**Table 4:** Categorical variables’ association with mean severity decrease. Cancer diagnoses were individually compared against the grand mean across all diagnoses

Variable	N	Mean	SD	*P*
Sex				
Female	596	3.51	2.62	
Male	466	3.34	2.68	0.29
Cancer history				
No	837	3.51	2.68	
Yes	196	3.01	2.34	0.016*
Cancer diagnosis				0.30
Bowel	124	3.69	2.47	0.247
Breast	211	3.59	2.68	0.33
Lung	188	3.33	2.81	0.58
Prostate	112	3.02	2.33	0.083
Other	423	3.42	2.66	0.97
Unemployment				
No	787	3.61	2.64	
Yes	275	2.95	2.60	0.00034***
Financial problems				
No	590	3.63	2.64	
Yes	472	3.20	2.63	0.0084**
Mental health problems				
No	891	3.51	2.67	
Yes	171	3.07	2.50	0.047*
Caring responsibilities				
No	855	3.47	2.64	
Yes	207	3.31	2.66	0.45
Mobility problems				
No	451	3.65	2.55	
Yes	611	3.28	2.70	0.024*

*indicates *p* < .05, **indicates *p* < .01, ***indicates *p* < .001

Age was associated with a lower concern severity at initial appointment, as was bowel cancer and being male. Deprivation, cancer history, unemployment, mental health problems, caring responsibilities, and mobility problems were all associated with a higher initial concern severity.

Number of days elapsed since initial appointment was associated with a larger decrease in concern severity, whereas cancer history, unemployment, financial problems, mental health problems, and mobility problems were associated with a smaller decrease in concern severity. Deprivation and having prostate cancer were near significance and were therefore also included in the multivariate analysis.

### 3.3 Multivariate analysis

To examine the predictors of initial concern severity, a multiple regression model (Model 1 in Table 5) was constructed with outcome variable “mean concern severity” and predictors gender, age, deprivation, cancer history, unemployment, breast cancer diagnosis, bowel cancer diagnosis, mental health problems, mobility problems, and caring responsibilities.

**Table 5:** Multiple linear regressions. In Model 1, a positive beta corresponds to an increase in initial concern severity. In Model 2, a positive beta corresponds to a larger reduction of concern severity at follow‐up

Variable	*b*	SE	95% CI	*P*
*Model 1: Initial concern severity*				
Sex (male)	−0.41	0.11	−0.62‐0.19	0.00026***
Age (1U = 10 y)	−0.76	0.050	−0.17‐0.0022	0.13
Deprivation	−0.028	0.011	−0.049‐0.0068	0.0098**
Cancer history	0.45	0.14	0.18‐0.72	0.00099***
Unemployment	0.36	0.14	0.083‐0.65	0.011*
Bowel cancer	−0.25	0.17	−0.59‐0.086	0.14
Mobility problems	0.33	0.12	0.10‐0.56	0.0044**
Mental health problems	0.24	0.16	−0.062‐0.55	0.12
Caring responsibilities	0.20	0.15	−0.098‐0.49	0.19
*Model 2: Severity change between assessments*				
Deprivation	−0.017	0.016	−0.049‐0.015	0.30
Cancer history	0.45	0.21	0.043‐0.86	0.030*
Unemployment	0.58	0.19	0.20‐0.96	0.0025**
Days elapsed (1U = 100 d)	−0.14	0.066	−0.26‐0.0058	0.041*
Prostate cancer	0.47	0.26	−0.039‐0.98	0.070
Mobility problems	0.30	0.17	−0.024‐0.62	0.069
Mental health problems	0.21	0.23	−0.23‐0.66	0.35

*indicates *p* < .05, **indicates *p* < .01, ***indicates *p* < .001

Gender and breast cancer were not independent (*χ* ^2^(1) = 355.7, *P* < 0.00001), and a model with breast cancer removed was not statistically different to a model containing both (*F*(1841,1842)=0.22, *P* = 0.64), whereas a model with gender removed was statistically different from a model containing both (*F*(1841,1842) = 10.22, *P* = 0.0014). Gender was retained in the model, and breast cancer was removed. No multicollinearity was observed, with variance inflation factors (VIF) being 1.4 or lower for all predictors. Multiple *R* ^2^ was 0.040, meaning that the model fit poorly, and explained a small fraction of variance of severity change.

To examine the predictors of change of concern severity between assessments, a multiple linear regression model (Model 2 in Table 5) was constructed with outcome variable mean difference in severity ratings and predictor number of days elapsed since initial assessment at review, deprivation, cancer history, unemployment, financial problems, mental health problems, mobility problems, prostate cancer. Financial problems and unemployment were not independent (*χ* ^2^(1) = 13.96, *P* = 0.00019), and a model with financial problems removed was not statistically different to a model containing both (*F*(1017,1018) = 3.22, *P* = 0.073), whereas a model with unemployment removed was statistically different from a model containing both (*F*(1017,1018) = 8.00, *P* = 0.0048). Consequently, unemployment was retained in the model and financial problems were removed. No multicollinearity was observed with VIF being 1.1 or lower for all predictors.

In the final severity change model, unemployment, cancer history and the number of days elapsed since initial assessment was significant predictors of change in severity ratings. Deprivation vigintile and mental health problems were not significant, and prostate cancer and mobility problems were near significance at *P* < 0.1. Multiple *R* ^2^ was 0.032, meaning that the model fit poorly, and explained a small fraction of variance of severity change.

## 4 DISCUSSION

The primary hypothesis was supported: there was a significant decrease in the mean severity of peoples’ concerns from initial assessment to follow‐up. Caveats to this finding will be discussed, but first, to understand the significance of the result it is important to understand the relationship between the HNA and the distress thermometer. As mentioned in the introduction, the HNA, the tool used to measure concern severity in this study was developed from the distress thermometer.^19^ They both use identical scoring methods, comprising a visual scale ranging from zero (no concern) to ten (maximum concern). The HNA uses this method to ascertain the severity of every individual concern, whereas the distress thermometer generates a single global distress score. Because the HNA focuses on the cause of distress rather than the level of it, it arguably provides assessors with clearer direction to prioritize the most serious concerns.

Having said this, there is a considerable body of work focused on identifying clinically meaningful cutoff scores on the distress thermometer. Values of 7 and above are indicative of “severe” distress, warranting referral to a psychologist.^27^, ^28^ Likewise, values below 4 are deemed “nonclinical”,^28^, ^29^, ^30^ meaning that people can manage their own distress in the main. It is not known if these well‐established cutoff scores on the distress thermometer meaningfully transfer to individual concerns on the HNA, but it is reasonable to suggest that they may. Recall that mean values reduced from 7.12 to 3.83, respectively, “severe” and “nonclinical” in distress thermometer literature. Psychometric work is planned to investigate the relationship between the measures, but based on these findings it is plausible to conclude that these patients went from severely concerned to significantly less so over the course of ICJ involvement.

Of course, ICJ was not the sole cause of the reduction. Several potential predictors of concern severity on initial assessment were identified. Being female, more deprived, having a history of cancer, being unemployed, and having mobility problems were all associated with a higher severity of concerns at initial appointment. Potential predictors of postintervention concern severity were also identified: improvement was reduced for unemployed people, for example, or those with a history of cancer. Time also played a part in recovery, suggesting that people naturally recover to a degree.^31^

Unemployment and cancer history showed the strongest relationships with initial severity and with change in severity at follow‐up. This makes sense as unemployment is associated with poor quality of life in general,^32^ and a strong predictor of depression, even where people have good social support and financial security.^33^ Likewise, a cancer history means the new diagnosis indicates either recurrence or further disease, both of which are known to be devastating.^34^

Other findings were harder to explain. For example, there is evidence that having mental health problems^35^ and caring responsibilities^36^ make managing a cancer diagnosis more difficult, so the null effects in both models were unexpected. In all, the low variance explained in both models points toward missing variables, suggesting that we did not measure the most relevant variables predictive of concern severity and/or the change of severity at follow‐up. It is hoped that the interventions that participants were referred to would explain more variance, and details of the interventions, patient participation, and patient satisfaction with intervention will be obtained for future evaluation. Other proposed measures include treatment outcomes, financial stability, and clinical prognosis.

Nevertheless, despite these caveats, this original, real‐world evaluation has shown that the holistic needs of a predominantly materially deprived cancer population can be met. The clinical and societal importance of this finding is difficult to overstate. Most cancer services recognize that people with cancer have complex needs, with some recognizing the financial impact of diagnosis,^2^ but we do not know of any other integrated, multiprofessional services that successfully meet these needs. As discussed, more work is needed to quantify the successful components of the service, but service user interviews have shown that the service is highly valued because it provides competent, consistent support at a traumatic time, allowing people to regain control of their lives.^37^ In line with the “navigator” systems used in some health services, the fact that users have one person to help guide them through a complex system is invaluable.^38^ It is well known that people who feel in control of their lives are much more likely to follow treatment guidelines,^39^ thereby reducing demand on emergency services and unplanned admissions.^40^

From the service perspective, early evaluation showed that ICJ functions through a combination of strong leadership overseeing a skilled workforce using an integrated system (HNA) that everybody understands and uses.^41^ Buy‐in across participating agencies at every level, but especially the highest level was essential.^42^ It is acknowledged that these are not easily replicable elements, but they can be replicated. Regardless of international context, poverty has a common impact. It prevents people getting the support they need. Any system successfully supporting individuals to live as well as they can, particularly at a time when all can seem lost, deserves particular attention.

## 5 LIMITATIONS

This was a single cohort pre‐ and poststudy. The change in concern severity cannot therefore be directly attributed to ICJ. People may have resolved these concerns by themselves over time. Much of the improvement seen could simply be a function of regression to the mean,^43^ especially as the people scoring the lowest on HNA on initial assessment were unlikely to have been formally followed up. In short, only those scoring high on the HNA to start with were followed up because they had the most serious concerns. While clinically appropriate, a statistical artifact of this is sample bias. By only analyzing those with high concern scores initially, the study inevitably increases its likelihood of demonstrating a large impact.

Despite originating from the distress thermometer, the HNA has not been subject to the same rigorous benchmarking and calibrating. Due to the HNA being so conceptually and visually similar it is reasonable to hypothesize that the cutoffs established in the distress thermometer would apply to the HNA, but this is not known. The HNA asks for each individual concern to be scored, whereas the distress thermometer asks for an overall score, so it is also highly likely that the scores would not always be equivalent. To examine the relationship in detail, future evaluations will add the distress thermometer to routine practice to examine the relationship between the instruments in a “real‐world” setting.

## 6 CONCLUSION

Interventions are required to support individuals affected by cancer. For example, effective assessment and care planning can lead to early intervention and improved outcomes. The impact of a cancer diagnosis is increasingly seen as wide ranging, yet the value of proactive services designed to meet wide‐ranging needs is hard to articulate to service commissioners. At a time where people expect more from their health and social services while budgets are simultaneously squeezed, it seems counterintuitive to go looking for even more problems to support.^44^ Yet, this study has led the way by showing that the wide‐ranging concerns that people diagnosed with cancer have, *can* be met by health and social care partners with an integrated community approach led by nonclinicians.^42^ Consequently, research that provides insight into initiatives that have successfully embedded integrated care can be used to inform colleagues across the cancer care profession.

One of the most striking findings was the high prevalence of financial concerns. The importance of mitigating the financial impact of cancer was recently discussed by Khera et al,^2^ who concluded that screening patients for financial distress were a “provocative idea.” This study has shown that in this cohort, it was an entirely sensible idea, alongside screening for a whole range of other needs as well. More data, including follow‐up measures of clinical outcomes and service usage, are required to better understand the appropriateness and impact of the referrals. Nevertheless, this article has shown for the first time the promising impact of a proactive multidisciplinary service led by nonclinicians.

## CONFLICT OF INTEREST

No conflict of interest disclosures from any authors.
