# Supplementary material for: Proactive community support tailored to holistic needs: A cohort study
Source: Cancer Med. 2018 Aug 13;7(9):4836–45. doi: 10.1002/cam4.1709 (PMC6144151; doi:10.1002/cam4.1709)
Supplement: Supplementary file 1 [file CAM4-7-4836-s001.zip › cam41709-sup-0001-supinfo]

ICJ routine data analysis paper for Cancer Medicine


# ICJ routine data analysis paper for Cancer Medicine

#### *Jan Savinc*

#### *originally run October 2017 - January 2018*

Data imported from file: ./data\_clean/ICJ\_routine\_data\_collated\_WIP\_2017-12-13.xlsx

## Data linking

To associate before-after differences in concern we need to first match up demographics & diagnosis info with Review data.

## Descriptives

A total of N=2413 cases were recorded between the start of the service in March 2014 and 21 July 2017 when the data was provided, of which there were N=2391 unique individuals.

The mean age was 63.5, SD=12.9, on a range of 22, 100.

The gender distribution was as follows:

| Gender | n | Proportion |
| --- | --- | --- |
| Female | 1286 | 53.29% |
| Male | 1127 | 46.71% |

The distribution of deprivation was as follows (Scottish Index of Multiple Deprivation, using SIMD 2016 data; 1 = most deprived)

| SIMD16\_Quintile | n | Proportion |
| --- | --- | --- |
| 1 | 1469 | 60.8% |
| 2 | 386 | 16.0% |
| 3 | 257 | 10.6% |
| 4 | 182 | 7.5% |
| 5 | 109 | 4.5% |
| NA | 14 | 0.6% |

| SIMD16\_Decile | n | Proportion |
| --- | --- | --- |
| 1 | 1050 | 43.4% |
| 2 | 419 | 17.3% |
| 3 | 223 | 9.2% |
| 4 | 163 | 6.7% |
| 5 | 141 | 5.8% |
| 6 | 116 | 4.8% |
| 7 | 74 | 3.1% |
| 8 | 108 | 4.5% |
| 9 | 65 | 2.7% |
| 10 | 44 | 1.8% |
| NA | 14 | 0.6% |

| SIMD16\_Vigintile | n | Proportion |
| --- | --- | --- |
| 1 | 724 | 30.0% |
| 2 | 326 | 13.5% |
| 3 | 288 | 11.9% |
| 4 | 131 | 5.4% |
| 5 | 144 | 6.0% |
| 6 | 79 | 3.3% |
| 7 | 62 | 2.6% |
| 8 | 101 | 4.2% |
| 9 | 65 | 2.7% |
| 10 | 76 | 3.1% |
| 11 | 61 | 2.5% |
| 12 | 55 | 2.3% |
| 13 | 35 | 1.4% |
| 14 | 39 | 1.6% |
| 15 | 52 | 2.2% |
| 16 | 56 | 2.3% |
| 17 | 38 | 1.6% |
| 18 | 27 | 1.1% |
| 19 | 22 | 0.9% |
| 20 | 22 | 0.9% |
| NA | 14 | 0.6% |

Marital status of individuals:

| Martial Status | n | Proportion |
| --- | --- | --- |
| Civil Partner | 12 | 0.5% |
| Divorced | 218 | 9.0% |
| Married | 941 | 39.0% |
| Partnered | 144 | 6.0% |
| Retired | 1 | 0.0% |
| Separated | 98 | 4.1% |
| Single | 520 | 21.5% |
| Unknown | 71 | 2.9% |
| Widowed | 408 | 16.9% |

Breakdown of ethnicities:

| Ethnicity | n | Proportion |
| --- | --- | --- |
| Any Mixed Background | 4 | 0.2% |
| Any Other Asian Background | 10 | 0.4% |
| Any Other Ethnic Background | 12 | 0.5% |
| Any Other White Background | 15 | 0.6% |
| Bangladeshi | 1 | 0.0% |
| Black African | 10 | 0.4% |
| Black Carribean | 1 | 0.0% |
| Chinese | 6 | 0.2% |
| Indian | 7 | 0.3% |
| Not Answered | 15 | 0.6% |
| Not Known | 22 | 0.9% |
| Pakistani | 18 | 0.7% |
| White Irish | 24 | 1.0% |
| White Other British | 32 | 1.3% |
| White Scottish | 1910 | 79.2% |
| NA | 326 | 13.5% |

Breakdown of emplyoment status:

| Employment Status | n | Proportion |
| --- | --- | --- |
| Employed | 506 | 21.0% |
| In education | 4 | 0.2% |
| Not answered | 21 | 0.9% |
| Retired | 1258 | 52.1% |
| Self Employed | 9 | 0.4% |
| Unemployed | 613 | 25.4% |
| Unknown | 2 | 0.1% |

Breakdown of ages by gender

#### Diagnosis groups

| CancerCategory4 | n | Proportion |
| --- | --- | --- |
| Bowel | 148 | 6.1% |
| Breast | 381 | 15.8% |
| Lung | 540 | 22.4% |
| Other | 1121 | 46.4% |
| Prostate | 226 | 9.4% |

#### Actual diagnoses

There were N=145 unique diagnoses under the *Primary Diagnosis* heading, and N=92 unique diagnoses under the *Primary Diagnosis Group* heading.

#### Breakdown of diagnoses by gender

Breakdown of main 4 diagnoses by gender

### Comorbidities

Number comorbidities reported:

| numComorbidities | n | Proportion |
| --- | --- | --- |
| 0 | 1115 | 46.2% |
| 1 | 629 | 26.0% |
| 2 | 366 | 15.1% |
| 3 | 211 | 8.7% |
| 4 | 79 | 3.3% |
| 5 | 16 | 0.7% |

#### Most common comorbidities

### What comorbidities were reported

Number of comorbidities reported, and proportion of individuals who reported them. Note that percentages don’t add up to 100% because people reported variable numbers of comorbidities. Only comorbidities with 10 or more cases shown, sorted by frequency

| Comorbidity | n | Proportion |
| --- | --- | --- |
| Arthritis | 337 | 25.9% |
| Diabetes | 229 | 17.6% |
| COPD | 228 | 17.5% |
| Hypertension | 228 | 17.5% |
| Mental Health | 199 | 15.3% |
| Asthma | 117 | 9.0% |
| Angina | 113 | 8.7% |
| Stroke | 101 | 7.8% |
| Osteoporosis | 95 | 7.3% |
| Heart Failure | 69 | 5.3% |
| Coronary Heart Disease | 63 | 4.8% |
| Musculoskeletal Problems | 62 | 4.8% |
| Emphysema | 49 | 3.8% |
| Cataracts | 33 | 2.5% |
| Hypothyroidism | 28 | 2.2% |
| Epilepsy | 24 | 1.8% |
| Musculoskeletal | 22 | 1.7% |
| Diverticular Disease | 19 | 1.5% |
| Ibs | 17 | 1.3% |
| Skin Complaints | 16 | 1.2% |
| Diverticular | 13 | 1.0% |
| Ulcerative Colitis | 11 | 0.8% |

#### Mental health

In addition to the reported comorbidities, mental health was also asked about at the start of the HNA when personal info was collected. Below is how many people reported having mental health issues:

| Mental Health Issues | n | Proportion |
| --- | --- | --- |
| no | 1984 | 82.2% |
| not answered | 76 | 3.1% |
| yes | 353 | 14.6% |

#### Financial issues

| Financial Diffculties | n | Proportion |
| --- | --- | --- |
| No | 1313 | 54.4% |
| Not Answered | 61 | 2.5% |
| Yes | 1039 | 43.1% |

#### Mobility issues

| Mobility Issues | n | Proportion |
| --- | --- | --- |
| No | 970 | 40.2% |
| Not Answered | 34 | 1.4% |
| Yes | 1408 | 58.4% |
| NA | 1 | 0.0% |

#### Literacy issues

| Any Literacy Issues | n | Proportion |
| --- | --- | --- |
| No | 2289 | 94.9% |
| Not Answered | 36 | 1.5% |
| Unknown | 1 | 0.0% |
| Yes | 87 | 3.6% |

#### Caring responsibilities

| Caring Responsibilites | n | Proportion |
| --- | --- | --- |
| No | 1974 | 81.8% |
| Not Answered | 42 | 1.7% |
| Yes | 397 | 16.5% |

#### Housing issues

Only a small set of N= individuals in 2017 were specifically asked about housing issues in the first stage of the assessment (separately from the Money/Housing concern in the HNA). Not very informative - very few people actually replied:

| Housing issue? | n | Proportion |
| --- | --- | --- |
| Adaptions required | 1 | 0.3% |
| maintenance issues | 3 | 0.9% |
| Neighbour issue | 3 | 0.9% |
| Not Answered | 309 | 94.8% |
| other | 6 | 1.8% |
| Property unsuitable due to location | 1 | 0.3% |
| property unsuitable following treatmen | 2 | 0.6% |
| Re- housing Necessary | 1 | 0.3% |

Number of comorbidities reported

### Stages in cancer journey

Follows the breakdown of cancer journey stages:

| ClientStage.clean | n | Proportion |
| --- | --- | --- |
| Living with condition | 399 | 16.5% |
| Palliative | 408 | 16.9% |
| Recently completed treatment | 179 | 7.4% |
| Recently diagnosed | 325 | 13.5% |
| Reoccurance | 34 | 1.4% |
| Undergoing tests | 108 | 4.5% |
| Undergoing treatment | 905 | 37.5% |
| NA | 58 | 2.4% |

```
## Scale for 'y' is already present. Adding another scale for 'y', which
## will replace the existing scale.
```

Distribution of stages in cancer journey

### Visit duration

The mean visit duration was 68.611809 minutes, SD=20.4855508

Distribution of durations of assessment

### Deprivation comparison between sample and Glasgow population estimate

Deprivation index comparison between sample (bars) and Glasgow population estimate (dashed line)

### Concerns identified

Total number of concerns identified in HNA, sorted by number of cases per concern.

# Initial assessment

The mean concern severity rating of all concerns raised at initial assessment was 6.13, SD=3.13, or a median of 7.

The mean number of concerns raised was 5.82, SD=5.02, or a median of 4

*Overall concern* at initial appointment was rated by N=1539 at a mean of 5.49, SD=3.34, or a median of 6

Overall concern at initial asssessment descriptives

| Mean | SD | N | SE |
| --- | --- | --- | --- |
| 5.73 | 3.28 | 1813 | 0.077 |

## Top 10 concerns at first assessment ranked by severity

The top 10 concerns ranked by severity at first assessment were as follows:

| concern | Mean | SD | N | SE |
| --- | --- | --- | --- | --- |
| Money or Housing | 7.31 | 2.74 | 1272 | 0.077 |
| Partner | 7.15 | 3.24 | 215 | 0.220 |
| Children | 7.10 | 3.35 | 222 | 0.220 |
| Worry/Fear/Anxiety | 6.97 | 2.81 | 600 | 0.110 |
| Work and Education | 6.81 | 2.99 | 143 | 0.250 |
| Caring Responsibilities | 6.74 | 3.39 | 193 | 0.240 |
| Unable to Express Feelings | 6.70 | 3.14 | 117 | 0.290 |
| Sadness or Depression | 6.67 | 3.01 | 355 | 0.160 |
| Getting Around | 6.59 | 2.92 | 669 | 0.110 |
| Other Relatives/Friends | 6.57 | 3.45 | 107 | 0.330 |

# Review scores, before & after

The number of people revisited for a review appointment was N=1146;

The mean scores were as below:

| Assessment | Mean | SD | N | SE |
| --- | --- | --- | --- | --- |
| 1st assessment | 7.12 | 2.50 | 4652 | 0.04 |
| Follow-up | 3.83 | 3.49 | 4474 | 0.05 |

The difference between mean of all concerns at initial appointment and at review was a significant reduction of 3.31, (95% CI 3.21–3.41); t=−64.683, df=4454.00, two-sided p=< .00001.

The initial mean of all concerns was 7.12, significantly higher than 7; t=3.156, df=4651.00, one-sided p=.00080. The follow up mean of all concerns was 3.83, significantly lower than 4; t=−3.204, df=4473.00, one-sided p=.00068.


## Characteristics of patients who did not have a Review assessment

There were N=1274 cases that only had a first assessment (HNA) and were not followed up, and were not also deceased or did not engage (N=159). It is not clear from the protocol whether there was a principled way of selecting individuals for follow-up; however, there was a significant difference at first appointment between mean severity of all concerns rated by individuals who only had a first appointment (M=6.37), and mean severity of all concerns rated by individuals who also had a follow-up (M=7.12), t=−9.682, df=2067.56, two-sided p=< .00001. Therefore we can claim post-factum that individuals with higher mean severity ratings were followed-up.

# Supplementary information

Below is a breakdown of which diagnostic groupings were used:

Note: where diagnosis is listed as ‘None’ or ‘Not answered’ there was a Primary Diagnosis but not a Primary Diagnosis Group or vice versa.

| Group | Primary Diagnosis | Primary Diagnosis Group |
| --- | --- | --- |
| Bowel | bowel; Bowel; Neo-endocryne tumour (bowel) | Bowel/Neo-endocryne; Colorectal; Coloretal |
| Breast | breast; Breast; Breast cancer; Pancreas | breast; Breast; Digestive Organs |
| Lung | lung; Lung; Lung Cancer | Lung; None |
| Other | Abdominal; Acute myeloid leukemia; Adenocarcinoma; ampullary; anal; Anal; Appendix; Astrocytoma; Basal cell carcinoma; Base of Skull; Bile duct; Bladder; Bone; Bone & Soft Tissue Sarcoma; Bone & Soft Tissue Sarcomas; Bone Marrow; brain; Brain; Bronchial carcinoma; Bronchial Carcinoma; Bronchus; Burketts Lymphoma; caecal; Caecal; Carcinoma; Carcinoma parotid & neck node; Carer; Cervical; Cervix; Cholangiocarcinoma; Cholongiocarcinoma; Chronic lymphocytic leukaemia; Colon; Colorectal; Corpus Uteri; Diffused Large B Cell Lymphoma; Disseminated Peritoneal Malignancy; Duodenal; Ear; Ear canal; Endometrial; Endometrium/Womb; Epiglottis; Essential thrombcythaemia; Ewings Sarcoma; Floor of mouth; Gall bladder; Gall Bladder; Gastric; Gastrointestinal; Gasttointestinal Stromal Tumour; Groin; Gullet; Gynecological; Head & Neck; Head and Neck; Hepatocellular carcinoma; Hepatocellular carcinomas; Hodgkin Lymphoma; Hodgkins Lymphoma; Intestine; Kidney; Larynx; Leukaemia; Lip; Liver; Liver cell; Lymphatic; lymphoma; Lymphoma; Mandible; Mandible, Oropharnx; MDS; Melanoma; Mesothelioma; Mouth; Multiple Myeloma; Multiple Myloma; myelodysplastic Syndrome; Myelodysplastic syndrome; Myeloma; Nasal; Nasel Cancer; Neck; neuroendocrine; Neuroendocrine; Neuroendocrine tumour; Non hodgkin lymphoma; Non Hodgkins Lymphoma; Non Small Cell Carcinoma; Not answered; Not Answered; Oesophageal; Oral; Oral cavity; Oral Cavity; Oropharynx; Other; Other (larynx); Other Cancer; Ovarian; Ovarian cancer; Pancreatic; Penile; Penis; Peritoneal; Peritoneum; Pharynx; Plasmacytoma; Polycythaemia rubra vera; Rectal; Rectum; Renal; Renal, pelvis and uterary; Rhabdomyosarcoma; Sacrcoma; Sarcoma; SCC Left Neck Cancer; SCC Right vocal cord; Sinonasal; Skin; Skin Cancer; Spindle Cell Sarcoma; Squamous cell carcinoma; Squamous Cell Carcinoma; Stomach; Supraglottic; supraglottis; Supraglottis; T2N0 Pyriform Sinus; T2NB Oropharynx; T4 Soft Palate Sqaumous Cell Carcinoma; Testicular; Throat; Thrombocythemia; thyroid; Thyroid; Tongue; Tonsil; Tounge; unknown primary; Unknown Primary; Urinary bladder; Uterine; Uterus; Vagina; Vaginal; Vocal cord carcinoma; Voicebox; Vulva; Womb | Abdomen; Adenocarcinoma; ampullary; anal; Anal; Astrocytoma; Bile duct; Bladder; Blod; Blood, bone marrow & lymphatic; Blood, Bone marrow, lymphatic; bone; Bone and soft tissue sarcomas; Brain; Brain & CNS; Brain and CNS; Bronchial carcinoma; caecal; Carcinoma; Carer; Cervical; Cholangiocarcinoma; Cholongiocarcinoma; Colorectal; Colortectal; Diffused Large B Cell Lymphoma; Digestive Organs; Endocrine glands; Endometrial; Endometrium/Womb; Essential thrombcythaemia; Ewings Sarcoma; female genital organs; Female genital organs; Gall bladder; Gastric; Gasttointestinal Stromal Tumour; Groin; Gynacological; Gynaecological; Haemotology; Head & Necj; Head & Neck; Head and Neck; Hepatocellular carcinoma; Hepatocellular carcinomas; Kidney; Leukaemia; Liver; Lung; lymphoma; Lymphoma; Male genital organs; mandible; MDS; Melanoma; Mesothelioma; Mouth; Myeloma; Nasal; Neuroendocrine; Not answered; Not Answered; Oesophageal; Oral cavity; Oral Cavity; Other; Other Cancer; Ovarian; Pancreatic; Peritoneal; Plasmacytoma; Polycythaemia rubra vera; Prostate; Renal; Renal, pelvis and uterary; Rhabdomyosarcoma; Sarcoma; SCC right vocal cord; Skin; Skin Cancer; Squamous cell carcinoma; Squamous Cell Carcinoma; Stomach; Supraglottic; Tongue; Tonsil; unknown primary; Unknown Primary; Urinary bladder; Urinary Tract; Urology; Vulva |
| Prostate | Prostate | Porstate; Prostate |
